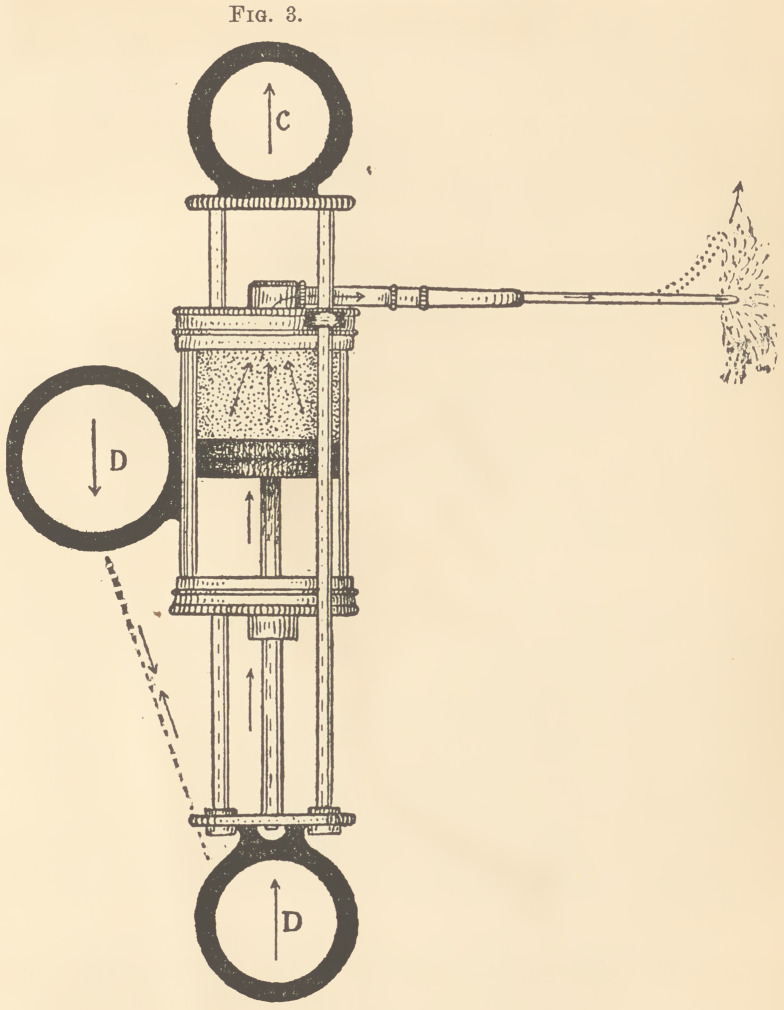# Spray-Syringe for Treatment of Diseases of the Antrum

**Published:** 1889-08

**Authors:** J. N. Farrar

**Affiliations:** New York City


### SPRAY-SYRINGE FOR TREATMENT OF DISEASES OF THE ANTRUM.

BY J. N. FARRAR, M.D., D.D.S., NEW YORK CITY.

SERIAL lectures, delivered by me early in the winter of 1878, before the students of the Pennsylvania College of Dental Surgery, on the "Radical and Heroic Treatment of Alveolar Abscess" and antral diseases, were, during the following year, published in the *Missouri Dental Journal*, and several of my instruments were shown. Since then, in the *Independent Practitioner*, and, still later, in the *Dental Cosmos*, my improvements upon the syringe used for treatment of the antrum (and *loculosis alveolaris*) have been illustrated and explained.

The object of the present article is to describe another syringe, which belonged to the original set.

In explaining the possible, yet infrequent, cure of an antral disease without extraction of an offending tooth, I referred to the normal condition of the antrum with its outlet upon the nasal cavity, situated a little above its floor, and said that "if the membranous coverings of the walls of these cavities are swollen, this natural outlet may be wholly or partly closed." Two such cases, at that time under my care, were cited, and I showed how, in each, the only means of discharge (especially if thick) was by using an aspirator which was therein described.

Figure 3 illustrates the syringe, which is made of metal, nickel-plated, and has a glass barrel. The oval end of the nozzle is closed like the end of an egg, but has several small jet-holes in its sides, about one-eighth of an inch from the apex. This closed end not only enables the nozzle to enter the antral chamber without irritation or pain, but it is also not liable to become clogged by being forced into contact with the tissues or walls of the antral cavity, which, when an ordinary tube is used, is generally the case.

Injury, likely to result from such violent pressure of the nozzle against the antral chamber or its thin roof (which is actually the floor of the orbit), is provided against by an adjustable bolt-gauge or collar, soldered to a sleeve, which slides backward and forward on the nozzle's body, and can be firmly set at any desired point, on the principle of the "universal clamp," as shown, in section, in Fig. 1, so that when the ball rests against the outward portal of

Fig. 1.

the drain-canal, the jets and spray can be made to fly in all directions from the central portion of the antral space, and with such force as to thoroughly drench and cleanse every part, as Fig. 2 exhibits.

These instruments were the first ever used, so far as I know, for spraying the antrum.

For home use of intelligent patients, who are capable of per-

Fig. 2.

forming this operation, I sometimes lend them the syringe above referred to, which is specially adapted for self-manipulation. It acts both as a syringe and an aspirator (Fig. 3.). Resting in the palm of the hand, by means of thumb and finger the piston can be made to play both ways. Notwithstanding its complicated appearance, the operation is really very simple. It should be borne in mind that a syringe, when used as an aspirator, should not be operated a second time without being cleaned, and never used upon another patient until it has been disinfected and the packing renewed.

The spray-nozzles that I now use for antral treatment are precisely the same as those that I used upon the syringes shown in 1879.

FIG. 3.

When the antrum is not accessible through the socket of a tooth, this spray-nozzle is replaced by a longer and slimmer tube to enter the antrum by way of the nares. These nasal tubes, of which there are two (right and left), are fitted directly upon the syringe-stub or interposed by a piece of elastic rubber tubing.

## Figures and Tables

**Fig. 1. f1:**
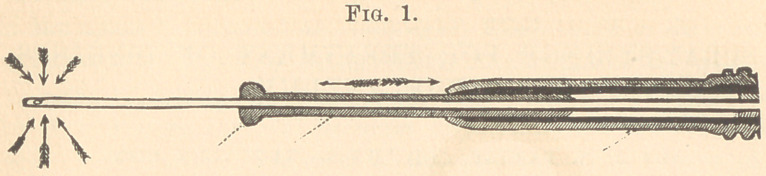


**Fig. 2. f2:**
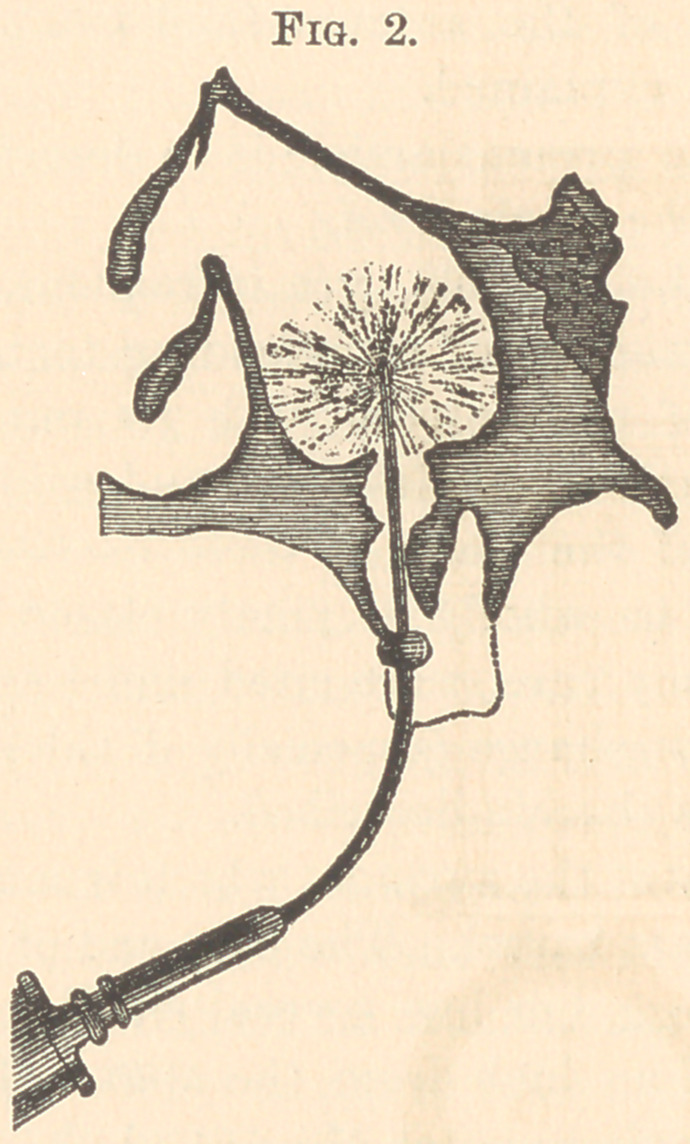


**Fig. 3. f3:**